# Parallel Evolution of Leukemic Clones in Myeloproliferative Neoplasms

**DOI:** 10.21203/rs.3.rs-8097141/v1

**Published:** 2025-11-17

**Authors:** Tyler M. Parsons, Aishwarya Krishnan, Infencia Xavier Raj, Andrew L. Young, David R. O’Leary, Jason Arand, Maggie Cox, Stephen T. Oh, Grant A. Challen

**Affiliations:** 1 Division of Oncology, Department of Medicine, Washington University School of Medicine; St. Louis, MO, USA, 63110.; 2 Division of Hematology, Department of Medicine, Washington University School of Medicine; St. Louis, MO, USA, 63110.

## Abstract

Myeloproliferative neoplasms (MPNs) are hematological diseases predominantly driven by the *JAK2*^V617F^ mutation. Progression from chronic-phase MPN to secondary acute myeloid leukemia (sAML) is a severe complication that dramatically worsens disease prognosis. While progression to sAML is classically linked to MPN clones acquiring additional cooperating mutations, the absence of the *JAK2*^V617F^ variant in some cases of sAML derived from *JAK2*^V617F^-mutant MPN suggests alternative mechanisms of transformation. Utilizing patient samples and *in vivo* modeling, we establish that leukemia-initiating clones driven by *TET2* mutations can emerge independently of *JAK2*-mutant cells and undergo positive selection in the pro-inflammatory MPN environment, leading to parallel disease evolution. Convergent profiling of mouse and human models identified IL-12 and TNFα as candidates providing extrinsic selective pressures and genetic and pharmacological inhibition of these cytokines mitigated the competitive advantage of *TET2*-mutant cells in an MPN background. These findings unveil therapeutic strategies to potentially prevent leukemic evolution in MPN patients by inhibiting specific cytokine signaling. Our data establish a new paradigm for clonal evolution of blood neoplasms by showing that disease progression in MPN can arise from parallel acute myeloid leukemia (pAML) clones independent of the primary disease.

## Introduction

Myeloproliferative neoplasms (MPNs) are a group of chronic hematological diseases driven by the acquisition of somatic mutations, predominantly *JAK2*^V617F^, in hematopoietic stem cells (HSCs)^1–3^. MPNs are characterized by the aberrant and unregulated proliferation of one or more myeloid lineages, resulting in the overproduction of mature hematopoietic cells in the bone marrow (BM) and peripheral blood (PB)^4–6^. This sustained overproduction manifests as polycythemia vera (PV; excess erythrocytes), essential thrombocythemia (ET; excess platelets), or myelofibrosis (MF; BM fibrosis). Consequently, patients face increased risks of blood viscosity and clotting, organ enlargement, joint pain and swelling, BM scarring, and are at-risk for progression to more aggressive disease states. The transformation of chronic-phase MPN to secondary acute myeloid leukemia (sAML) is a severe complication traditionally linked to the acquisition of additional mutations in the MPN driver clone in genes encoding epigenetic regulators (e.g. *TET2*, *DNMT3A*, *ASXL1*), tumor suppressors (e.g. *TP53*, *JARID2*) and signaling molecules (e.g. *N*/*K*-*RAS*)^7–10^. However, the *JAK2*^V617F^ driver mutation is occasionally absent in sAML that transforms from antecedent *JAK2*-mutant MPNs, which suggests alternative mechanisms of disease evolution^11^. Proposed explanations for the absence of the *JAK2*^V617F^ mutation at the sAML stage include somatic reversion and loss of heterozygosity, but an underexplored possibility is that sAML originates from independent clones that evolve in parallel to the MPN during the natural history of clonal hematopoiesis (CH)^12,13^.

Here, we establish that pre-leukemic clones can arise independently of the MPN and outcompete the *JAK2*^V617F^-mutant cells to manifest disease progression, challenging the traditional dogma of post-MPN sAML evolution. We leveraged a series of primary patient samples **(Table S1)** and pre-clinical models to investigate the growth and evolution of parallel clones in an MPN background. We demonstrate that independent clones carrying *TET2* and *TP53* mutations are positively selected by MPN-derived pro-inflammatory cytokines such as IL-12 and TNFα. Importantly, we demonstrate these mechanisms are amenable to targeted therapy as genetic and pharmacological inhibition of IL-12- and TNFα-signaling ameliorates the competitive advantage of *TET2*-mutant cells in the presence of *JAK2*^V617F^-mutant MPN. Collectively, these data demonstrate that *JAK2*^V617F^-mutant cells condition an environment that confers a selective advantage for the parallel growth of independent clones in the background of existing MPN. In such cases, the more accurate diagnosis is two independent diseases evolving in the same patient – the primary MPN and a parallel acute myeloid leukemia (pAML). Improved understanding of the phylogeny of MPN disease evolution may offer new clinical opportunities for these patients who currently have very limited treatment options.

## Main Text

### Leukemic Clones Can Arise Independently and Undergo Positive Selection in a Background of MPN

**Figure 1.**
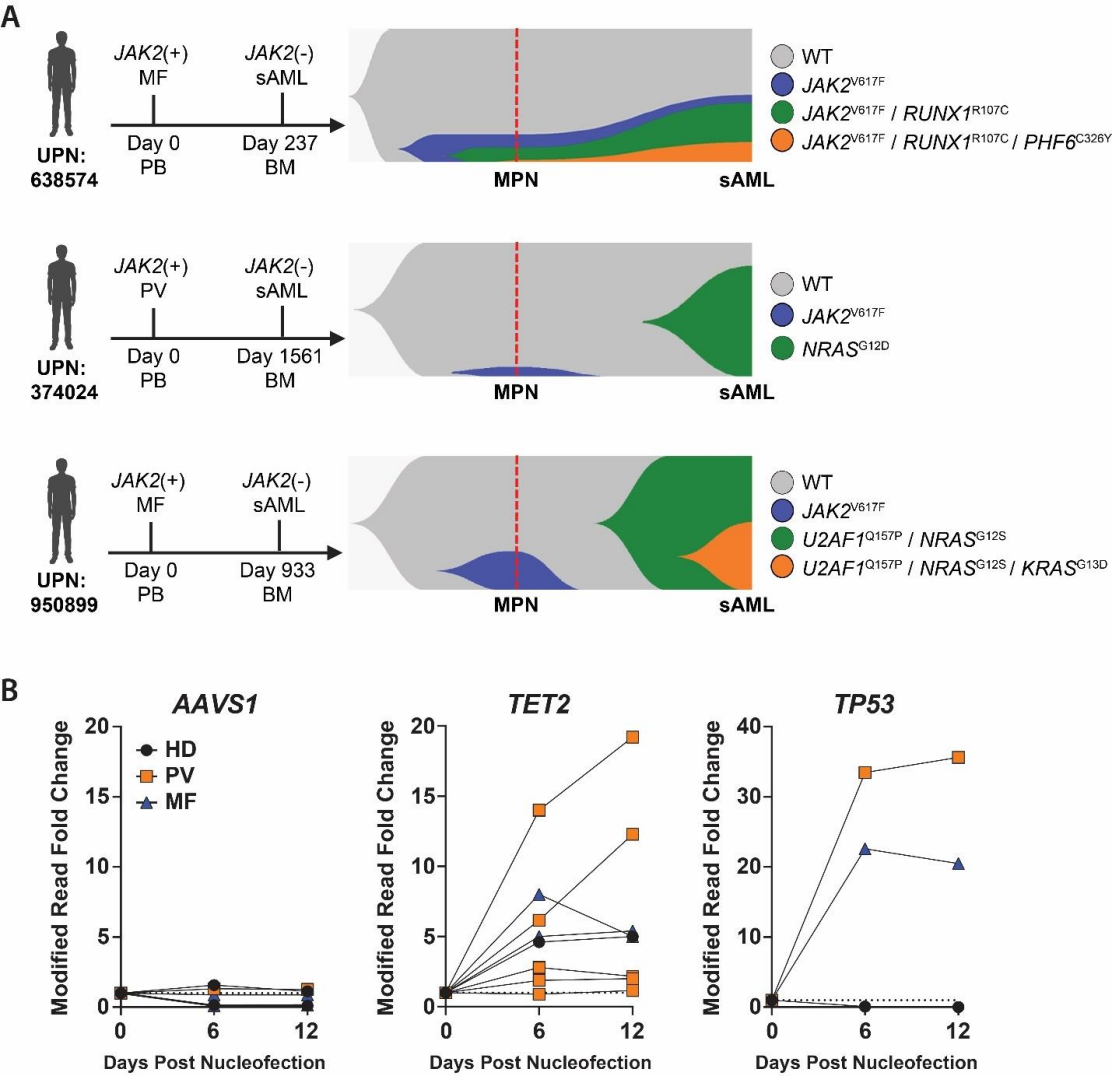
Leukemic Clones Can Arise Independently and Undergo Positive Selection in a Background of MPN. **(A)** Clonal hierarchy visualization of three paired (MPN and sAML) patient samples resolved by single-cell genomic sequencing with the x-axis expressed as time and the y-axis displaying proportionate prevalence of each clone within the population. **(B)** *ex vivo* competition assay showing VAF of engineered *AAVS1*, *TET2*, and *TP53* mutations in cord blood CD34^+^ cells in co-culture with CD34^+^ cells from *JAK2*^V617F^ PV or MF patients or healthy donor (HD) BM.

To investigate the clonal trajectory post-MPN sAML, we performed single cell genomic analysis for three paired patient samples (2 MF; 1 PV) who progressed from a *JAK2*^V617F^-mutant MPN to a *JAK2*-negative sAML as defined by clinical sequencing **(Figure S1A)**. Single-cell sequencing data from purified CD34^+^ cells for two patients (MF UPN:950899; PV UPN:374024) demonstrated that mutations detected at the sAML stage were not detected within the *JAK2*-mutant clones at the MPN stage (Figure 1A). Despite clinical genomics defining MF UPN:638574 as *JAK2-*negative sAML, single cell analysis of purified CD34^+^ cells clearly showed the leukemic mutations were acquired in the founding MPN clone (Figure 1A). This was confirmed by droplet digital PCR (ddPCR) wherein the *JAK2*^V617F^ mutation was detected at both the MPN and the sAML stages **(Figure S1B)**. While these data show there are multiple routes to transformation of post-MPN sAML, the single cell genomics clearly show that the leukemia-initiating mutations are not always present within the *JAK2*-mutant clones and can arise independently and outcompete the MPN cells to drive transformation.

Given this finding, we developed an *ex vivo* competition system to quantify the proliferation of independent clones in the presence of MPN cells utilizing CRISPR/Cas9 editing. 1×10^5^ CD34^+^ cells from *JAK2*-mutant MPN patients or healthy donor (HD) BM were co-cultured for 12-days with 2×10^4^ cord blood (CB)-derived CD34^+^ cells nucleofected with gRNAs targeting *AAVS1*, *TET2* or *TP53*. CRISPR edited alleles were tracked via targeted genomic sequencing at 6-day intervals and compared to initial values 24-hours post-nucleofection. In the presence of MPN patient CD34^+^ cells, *TET2*- and *TP53*-mutant clones expanded significantly more compared to co-culture of the same clones with HD BM control CD34^+^ cells (Figure 1B). Interestingly, PV patient cells supported the growth of both *TET2*- and *TP53*-mutant cells moreso than MF patient cells, a finding consistent with clinical reports that the *JAK2*-mutant MPN to *JAK2* wild-type sAML trajectory is more common in PV patients^11,13^.

### *JAK2*^V617F^-Mutant MPN Accelerates Expansion of Independent *TET2*- and *TP53*-Mutant Clones

**Figure 2.**
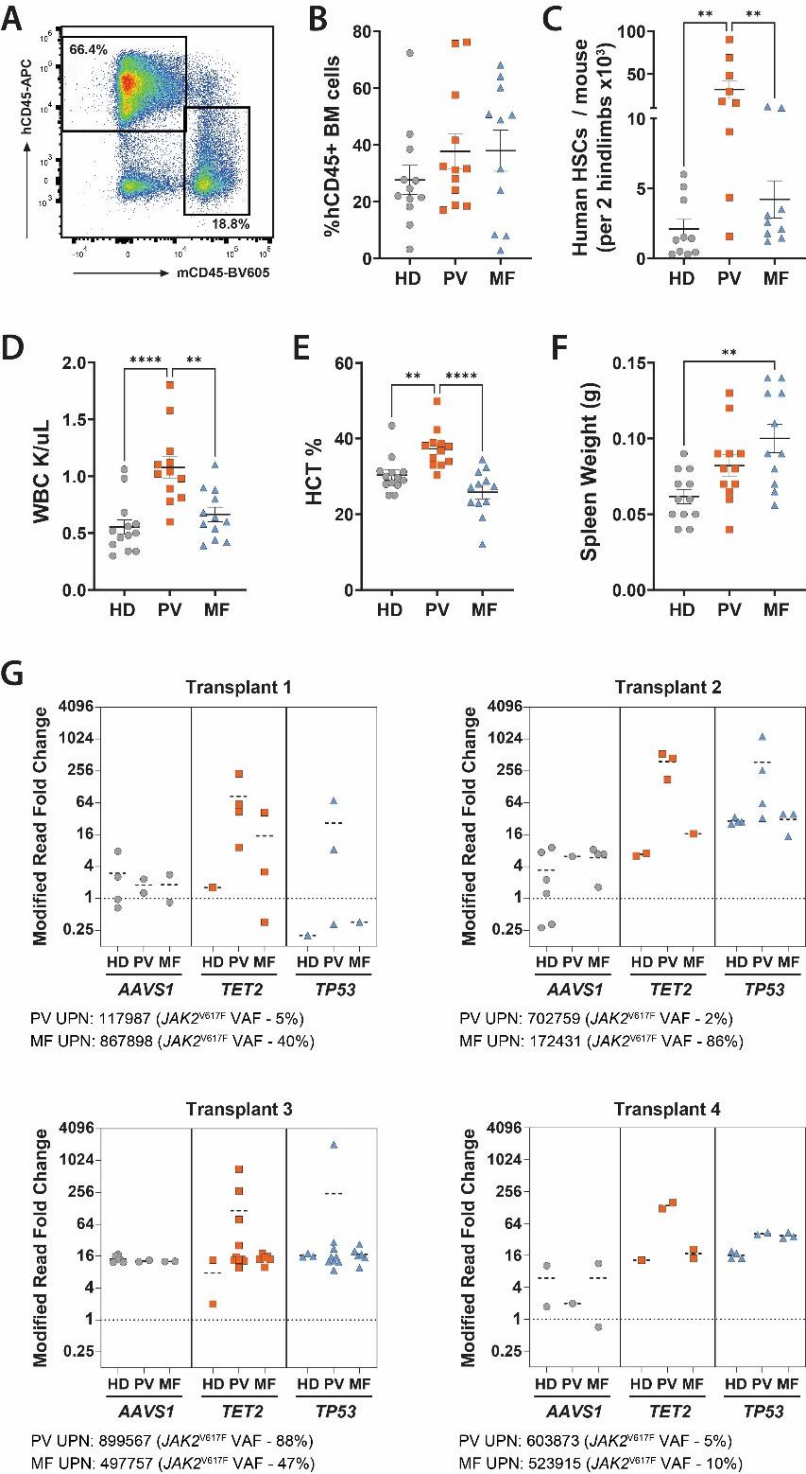
*JAK2*^V617F^-Mutant MPN Accelerates Expansion of Independent *TET2*- and *TP53-*Mutant Clones. **(A)** Representative flow cytometry plot depicting human cell engraftment in BM of NSGS mice. **(B)** Quantification of overall hCD45+ BM engraftment, **(C)** human HSC number, **(D)** white blood cell count, **(E)** hematocrit and **(F)** spleen weights compiled across PDX experiments. **(G)** Relative fold change of CRISPR modified reads in hCD45+ BM cells 16-weeks post-transplant compared to day 0 values of tracked *AAVS1*, *TET2*, and *TP53* engineered mutations in cord-blood CD34^+^ cells (n= 12 recipient mice sourced from 4 separate donors for HD, PV, and MF). **p ≤0.01, ****p ≤0.0001

To explore competition dynamics between MPN cells and independent clones, we leveraged our MPN patient derived xenograft (PDX) system^14^. *TET2* and *TP53* mutations were engineered into CB-derived CD34^+^ cells using CRISPR/Cas9 homology-directed repair (HDR) to introduce *TET2*^1216*^ and *TP53*^R248Q^ knock-in (KI) mutations into CB-derived CD34^+^ cells **(Figure S2)**. These mutations were chosen from the limited literature describing *TET2* and *TP53* mutations as the most common variants in *JAK2*-negative post-MPN sAML^15,16^. A single-stranded oligo donor nucleotide (ssODN) was designed to introduce a silent mutation in the inert *AAVS1* locus to serve as a trackable negative control genetic barcode. As most *TET2* and *TP53* mutations in myeloid malignancies lead to loss of function^17–22^, indels resulting from CRISPR/Cas9 cutting but failed ssODN directed repair were additionally tracked. These edits should also provide the cells with a competitive advantage and increase the clonal complexity within a given experiment. PDX models were established by co-transplanting 2.0×10^4^ cells from each of the *AAVS1*, *TET2* and *TP53* nucleofected populations into NSGS immunodeficient mice with 1.0×10^5^ CD34^+^ cells derived from either HD BM confirmed to be *JAK2*^V617F^-negative by ddPCR (control; n=4), *JAK2*-mutant MF (n=4), or *JAK2*-mutant PV (n=4) patients **(Table S1)**. Flow cytometric analysis was performed to confirm PB lineage reconstitution **(Figure S3)**, and PDX models supported robust engraftment of human cells (Figure 2A) in the BM (Figure 2B). Notably, mice engrafted with PV patient cells supported significant expansion of human HSCs (mCD45− hCD45+ LINEAGE− CD34+ CD38− CD45RA− CD90+) in the BM (Figure 2C). PDXs recapitulated classical phenotypes of the patients from which they were derived (WBC, HCT, splenomegaly; Figure 2D–F) and served as a relevant platform to observe how different MPN subtypes support the expansion of independent *TET2*- and *TP53*-mutant clones. Targeted genomic sequencing was performed on hCD45+ cells isolated from the BM of recipient mice at the conclusion of each independent transplant (16-weeks) to quantify gRNA-mediated variant allele fraction (VAF). These data were compared to pre-transplant values to analyze the growth dynamics of parallel clones isolated from a *JAK2*^V617F^-mutant MPN-derived PDX host in comparison to the same pool of edited cells in a HD control host. MF and PV patient cells supported the growth of *TET2-* and *TP53-*mutant clones significantly more than HD control BM across four separate experiments established with independent starting MPN patient material (Figure 2G). Notably, cohorts established from PV patient cells exhibited the highest expansion of independent clones, particularly for *TET2*-mutant cells, mirroring results from the *ex vivo* experiment. These data highlight the robust expansion capacity of *TET2*- and *TP53*-mutant clones within *JAK2*^V617F^-mutant MPN environments *in vivo*.

### Low Burden of *Jak2*^V617F^-Mutant Cells Specifically Supports Parallel Expansion of *Tet2*-Mutant Clones

With the finding that independent *TET2* and *TP53* clones display a significant growth advantage in a *JAK2*^V617F^-mutant environment, we established murine chimera models to provide a platform for mechanistic studies. These systems allow for precise control of the burden of each mutant cell population at the start of each transplant, facilitating a more detailed study of clone behavior and interaction than afforded by PDX models. We utilized mice with inducible expression of *Jak2*^V617F^ in the hematopoietic system (Vav-Cre;*Jak2*^V617F/+^ = “*Jak2*^V617F^”), which generate a PV-like phenotype, or wild-type (WT) control mice as a host background and *Tet2* heterozygous loss-of-function (Vav-Cre;*Tet2*^fl/+^ = “*Tet2*^Δ/+^”) or *Tp53* heterozygous knock-in (*Tp53*^R172H/+^) mice as the competing test populations. 2.5×10^6^ BM cells from either *Jak2*^V617F^ or WT control mice (CD45.2) were mixed with 5×10^5^ BM cells from either *Tet2*^Δ/+^, *Tp53*^R172H/+^, or WT test BM (CD45.1/2) and transplanted into lethally irradiated recipients (CD45.1) to produce a starting fraction of “test” BM of approximately 15%.

**Figure 3.**
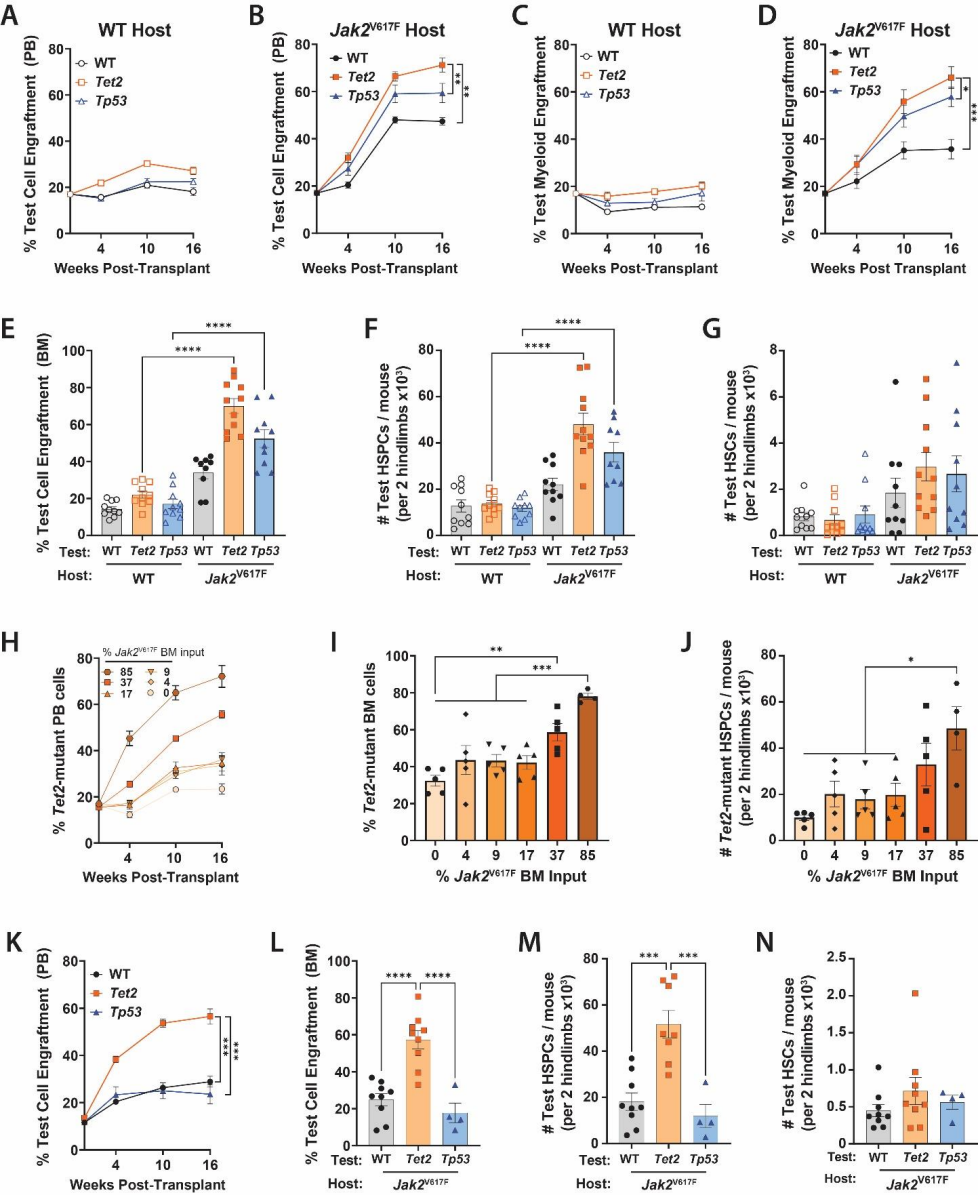
Low Burden of *Jak2*^V617F^-Mutant Cells Specifically Supports Parallel Expansion of *Tet2*-Mutant Clones. **(A**, **B)** Peripheral blood engraftment of WT control, *Tet2*^Δ/+^ and *Tp53*^R172H/+^ test cells in (A) WT or (B) a *Jak2*^V617F^ host BM. **(C**, **D)** PB myeloid (Gr-1^+^ Cd11b^+^) engraftment of WT control, *Tet2*^Δ/+^ and *Tp53*^R172H/+^ test cells in (C) WT or (D) *Jak2*^V617F^ host BM. **(E-G)** Quantification of WT, *Tet2*^Δ/+^, *Tp53*^R172H/+^ test cell (E) BM engraftment, (F) HSPC (Lineage^−^ Sca-1^+^ c-Kit^+^) number and (G) HSC (Lineage^−^ Sca-1^+^ c-Kit^+^ CD48^−^ CD150^+^) number in the BM of recipient mice 18-weeks post-transplant (n =10 / group). **(H-J)** Quantification of WT control and *Tet2*^Δ/+^ cell (H) PB engraftment, (I) BM engraftment and (J) HSPC numbers in recipient mice transplanted with indicated fractions of *Jak2*^V617F^ BM input cells (n = 5 / group). **(K-N)** Quantification of WT control, *Tet2*^Δ/+^, *Tp53*^R172H/+^ test cell (K) PB engraftment, (L) BM engraftment, (M) HSPC numbers and (N) HSC numbers in recipient mice transplanted with 50% *Jak2*^V617F^ BM input (n= 5–9 / group). *p ≤0.05, **p ≤0.01, ***p ≤0.001, ****p ≤0.0001

Flow cytometric analysis was performed to evaluate PB lineage reconstitution **(Figure S4)**. By 16-weeks post-transplant, *Jak2*^V617F^-mutant host cells significantly supported expansion of both *Tet2*^Δ/+^ and *Tp53*^R172H/+^ test populations in the PB compared to a WT control host background (Figure 3A–B). However, the WT test population also exhibited an engraftment increase in the presence of *Jak2*^V617F^-mutant cells (Figure 3B). This engraftment advantage was largely restricted to peripheral lymphoid cells, consistent with prior reports that *JAK2*^V617F^ mutations confer lymphoid deficiency. Within the PB myeloid compartment in a *Jak2*^V617F^-mutant background, the WT test population exhibited significantly reduced chimerism compared to *Tet2*^Δ/+^ and *Tp53*^R172H/+^ test populations (Figure 3C–D). BM analysis 18-weeks post-transplant revealed a similar competitive advantage of *Tet2*^Δ/+^ and *Tp53*^R172H/+^ cells in a *Jak2*^V617F^-mutant background (Figure 3E). Interestingly, the effect was most pronounced in the hematopoietic stem/progenitor cell (HSPC; c-Kit+ Sca-1+ Lineage- “KSL”) compartment rather than the most primitive long-term hematopoietic stem cells (HSCs; KSL CD48− CD150+) (Figure 3F–G).

As *Tet2*-mutant clones demonstrated an enhanced growth advantage in a *Jak2*^V617F^-environment compared to *Tp53*-mutant clones in both PDX and murine chimeras, we aimed to determine the minimum *Jak2*^V617F^-mutant cell burden required to support parallel *Tet2*-mutant clone expansion. A titration experiment was performed wherein BM cells from *Jak2*^V617F^ mice were diluted at predetermined ratios with WT BM while maintaining a fixed *Tet2*^Δ/+^ cell input (15%). There was a threshold effect between starting *Jak2*^V617F^ cell input and the conferred competitive advantage of *Tet2*^Δ/+^ cells with a ~35% *Jak2*^V617F^ mutant cell burden being the threshold needed to support robust expansion of *Tet2*^Δ/+^ cells (Figure 3H–J).

Based on these data, we established chimeras with 50% *Jak2*^V617F^-mutant BM, 35% WT support BM, and 15% *Tet2*^Δ/+^, *Tp53*^R172H/+^ or WT test BM to determine if this was also sufficient to induce outgrowth of parallel *Tp53*-mutant cells. As anticipated, a 50% starting *Jak2*-input supported positive selection of *Tet2*^Δ/+^ cells in the PB, BM, and HSPC compartments. However, this *Jak2*^V617F^-mutant burden did not support engraftment of *Tp53*^R172H/+^ or WT cells unlike the higher (85%) *Jak2*^V617F^-mutant cell input (Figure 3K–M). These findings highlight a specific interaction between *Jak2*^V617F^-mutant MPN cells and independent *Tet2*-mutant clones.

### IL-12 and TNFα Drive Expansion of *Tet2*-Mutant Clones in a *Jak2*^V617F^-Mutant Environment

**Figure 4.**
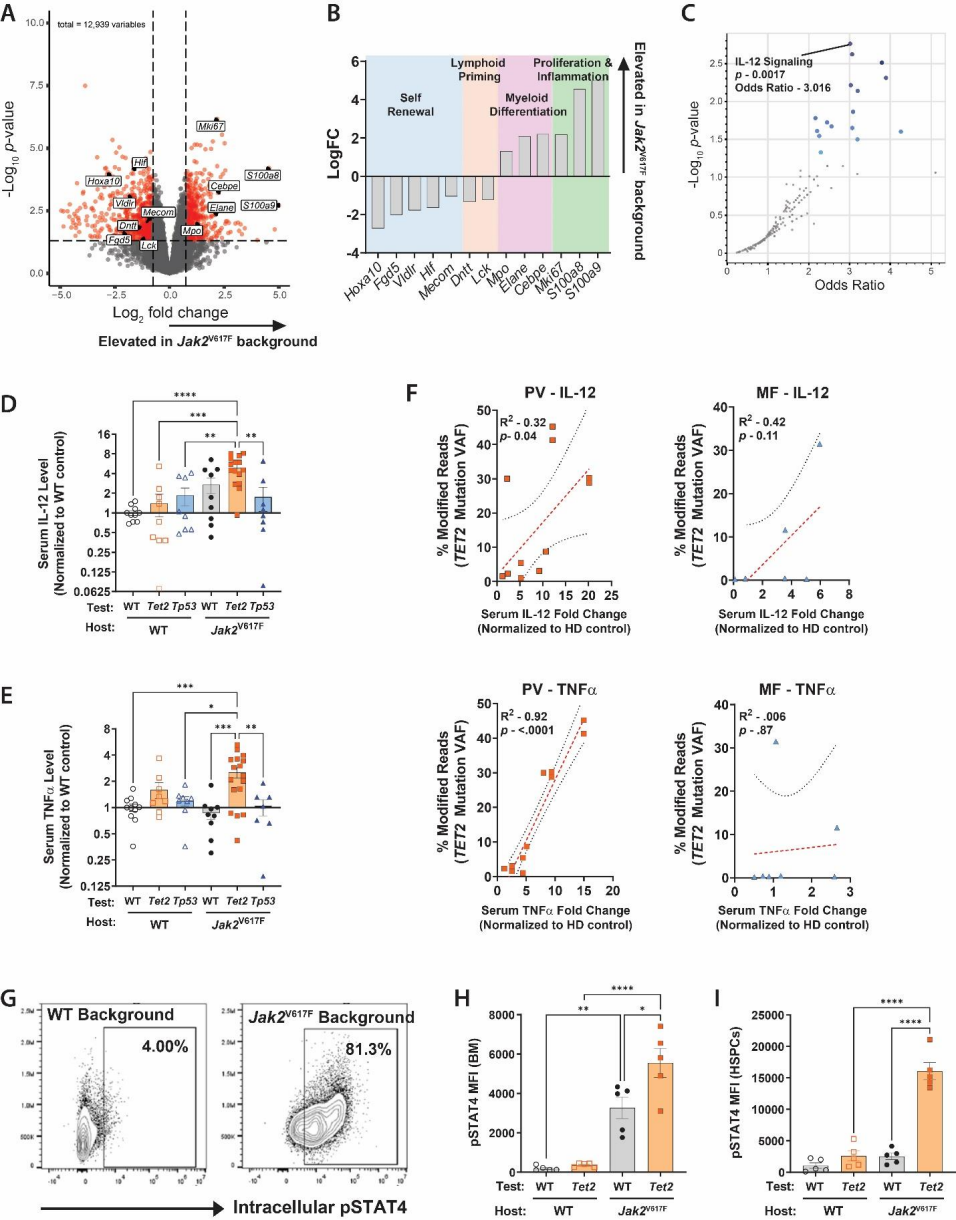
IL-12 and TNFα Drive Expansion of *Tet2*-Mutant Clones in a *Jak2*^V617F^-Mutant Environment **(A)** Volcano plot showing differentially expressed genes between *Tet2*^Δ/+^ HSPCs from a *Jak2*^V617F^ environment compared to *Tet2*^Δ/+^ HSPCs from a WT environment. **(B)** LogFC values for *Tet2*^Δ/+^ HSPCs DEGs from indicated pathways. **(C)** Volcano plot of gene set over-representation analysis (ORA) identifying IL-12 signaling as the most significantly enriched pathway in *Tet2*^Δ/+^ HSPCs from a *Jak2*^V617F^ environment. **(D-E)** Serum cytokine levels (normalized to WT control chimeras) of (D) IL-12 and (E) TNFα from murine mixed chimeras (n= 9–15 / group). (**F**) Serum cytokine fold change (normalized to HD control mice) for PV and MF PDXs plotted against relative increase in VAF of individually tracked *TET2* mutations engineered into cord blood CD34^+^ cells (n= 12 recipient mice sourced from 4 separate donors for each HD, PV, and MF). **(G)** Representative flow cytometry plots showing intracellular pSTAT4 staining in *Tet2*^Δ/+^ BM cells in WT or *Jak2*^V617F^ backgrounds. **(H-I)** Median fluorescent intensity (MFI) of intracellular pSTAT4 levels in (H) whole BM and (I) HSPCs (n = 5 / group). *p ≤0.05, **p ≤0.01, ***p ≤0.001, ****p ≤0.0001

As our data show a particularly strong selection of independent *Tet2*-mutant clones in an MPN background, we focused mechanistic studies to define this relationship. As the selective advantage for *Tet2*^Δ/+^ cells in and MPN background was most evident at the level of HSPCs, gene expression profiling was performed by RNA-sequencing analysis of *Tet2*^Δ/+^ HSPCs (CD45.1/2+ c-Kit+ Sca-1+ Lineage-) isolated from either WT or *Jak2*^V617F^-mutant hosts 18-weeks post-transplant. Analysis of differentially expressed genes (DEGs; Figure 4A) demonstrated that *Tet2*^Δ/+^ HSPCs from a *Jak2*^V617F^-mutant environment exhibited a pronounced proliferation bias, exemplified by upregulation of *Mki67*, and increased expression of pro-myeloid differentiation genes such as *Elane*, *Mpo*, and *Cebpe*. The inflammatory response was also heightened with elevated levels of *S100a8* and *S100a9* which function as key promoters of myeloid inflammation and are routinely upregulated in HSPCs in the contexts of myeloid skewing and chronic inflammatory states^23^. This myeloid differentiation bias occurred at the expense of lymphoid priming marked by decreased expression of *Dntt* and *Lck*, essential genes for lymphoid lineage commitment. Genes classically associated with HSC identity, such as *Hoxa10*, *Fgd5*, *Vldlr*, *Hlf*, and *Mecom*, were also downregulated in *Tet2*^Δ/+^ HSPCs from a *Jak2*^V617F^-mutant environment (Figure 4B), suggesting these cells were being pushed towards proliferation at the expense of self-renewal. Collectively, these findings indicate that a *Jak2*^V617F^-mutant environment drives *Tet2-*mutant HSPCs toward a proliferative, pro-myeloid, inflammatory phenotype. We next interrogated whether the gene expression changes observed in *Tet2*^Δ/+^ HSPCs from a *Jak2*^V617F^-mutant environment were due to specific effects on *Tet2*^Δ/+^ cells and not a general effect for any HSPCs exposed to MPN cells by RNA-seq comparison of *Tet2*^Δ/+^ and WT HSPCs isolated from a *Jak2*^V617F^-mutant environment. Differential expression analysis confirmed that the transcriptomic alterations previously identified in *Tet2*^Δ/+^ HSPCs remained dysregulated in *Tet2*^Δ/+^ HSPCs compared to WT HSPCs from a *Jak2*^V617F^-mutant environment **(Figure S5)**. These findings demonstrate that the identified gene dysregulation is unique to *Tet2*-mutant cells within a *Jak2*^V617F^-mutant milieu and not a generalized feature of HSPCs exposed to MPN cells.

To discern the most dysregulated signaling pathways that may be driving the observed transcriptional changes, over-representation analysis (ORA) was performed on the identified DEGs. ORA identified IL-12-mediated signaling as the most significantly dysregulated cancer-related pathway between *Tet2*-mutant HSPCs isolated from a *Jak2*^V617F^-mutant background compared to from a WT background (Figure 4C). To further classify over-represented pathways, DEGs were quantitatively scored based on how frequently they appear in a cancer-related pathway relative to their frequency across all cancer-related pathways and used to weight gene set terms by their relevance across the dataset. Uniform manifold approximation and projection (UMAP) was then applied for dimensionality reduction to visualize relationships between enriched pathways. Again, among the identified clusters, IL-12-mediated signaling emerged as the most highly enriched pathway. Adjacent points in the UMAP space, inferring biological relevance of over-represented gene sets, included TNF receptor signaling and IL-12 signaling mediated by STAT4, highlighting these related signaling pathways as potential selection factors for *Tet2*^Δ/+^ HSPC expansion in a *Jak2*^V617F^-mutant environment **(Figure S6)**.

It is well-documented that MPN cells secrete high levels of many pro-inflammatory cytokines^24–27^. Moreover, we and others have shown that many common CH mutations impart growth advantages to the mutant clones under different conditions of inflammation^28–32^. To determine if the observed gene expression changes in *Tet2*^Δ/+^ HSPCs in a *Jak2*^V617F^-mutant environment were associated with altered cytokine levels due to the MPN cells, global cytokine profiling of PB serum from murine chimera and PDX experiments was performed. The overlap of cytokines increased in MPN models compared to relevant control comparators revealed four candidates - IL-1, IL-12, IL-27, and TNFα **(Figure S7A)**. We sought to determine if any of these cytokines might create a competitive advantage for *TET2*-mutant clones. CB-derived CD34^+^ cells were CRISPR/Cas9-engineered with *TET2* mutations and cultured with each candidate cytokine at concentrations ranging from 1–100 ng/mL for 6-days. TNFα and IL-12 were the most effective in accelerating *TET2*-mutant cell proliferation *in vitro* **(Figure S7B)**. Moreover, from *in vivo* studies, increasing IL-12 and TNFα levels correlated with *TET2*-mutant clonal expansion in murine (Figure 4D,E) and PDX transplants (Figure 4F). This effect was most pronounced in PDX mice established from PV patient cells (Figure 4F). To determine the source of the aberrant signaling, IL-12 and TNFα levels were measured in serum from transplant donor mice of each genotype. IL-12 levels were significantly elevated in *Jak2*^V617F^-mutant mice compared to WT and *Tet2*^Δ/+^ donor mice. Conversely, TNFα was significantly elevated in *Tet2*^Δ/+^ donor mice **(Figure S8)**. Notably, TNFα has been reported to correlate with *TET2*-mutant CH and IL-12 is a known TNFα stimulator that is elevated in MPN patients^33–38^. This suggests a model whereby IL-12 secreted by MPN cells induces TNFa over-production by *Tet2*-mutant cells to condition an environment that fosters their development.

We hypothesized that IL-12 secreted by MPN cells directly acts upon *TET2*-mutant cells to phosphorylate STAT4, a key mediator of IL-12 signaling^39–41^. Intracellular flow cytometry analysis (Figure 4G) revealed that pSTAT4 levels were elevated in *Tet2*^Δ/+^ BM cells isolated from a *Jak2*^V617F^-mutant background 10-weeks post-transplant compared to those isolated from a WT background or from WT cells from either background (Figure 4H). Strikingly, the pSTAT4 elevation was most significant in the *Tet2*^Δ/+^ HSPC population isolated from a *Jak2*^V617F^-mutant environment (Figure 4I). This indicates a potential mechanistic link between IL-12 signaling and *Tet2*^Δ/+^ HSPC proliferation in a *Jak2*^V617F^-mutant context, suggesting that targeting this pathway could mitigate the competitive advantage in this genetic setting.

### Inhibition of Inflammatory Cytokines Mitigates the Competitive Advantage of *TET2*-Mutant Cells in a *Jak2*-Mutant Environment

**Figure 5.**
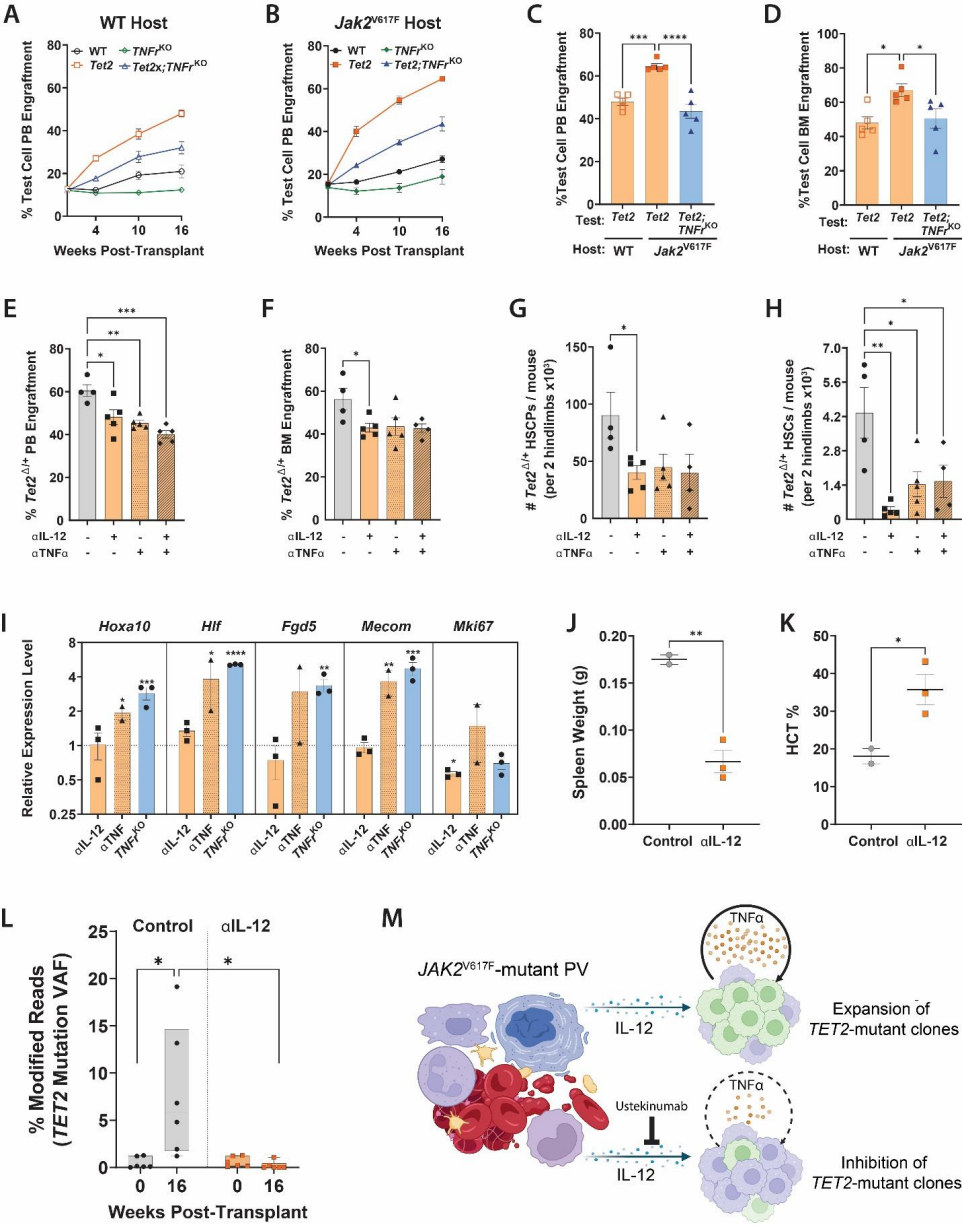
Inhibition of Inflammatory Cytokines Mitigates the Competitive Advantage of *TET2*-Mutant Cells in a *Jak2*-Mutant Environment. **(A**, **B)** Peripheral blood engraftment of WT control, *Tet2*^Δ/+^, *TNFr*^KO^ and *Tet2*^Δ/+^;*TNFr*^KO^, test cells in (A) WT or (B) a *Jak2*^V617F^ host BM (n= 5 / group). (**C**) 16-week peripheral blood engraftment of *Tet2*^Δ/+^ and *Tet2*^Δ/+^, *TNFr*^KO^ test cells in WT or *Jak2*^V617F^ host BM (n= 5 / group). (**D**) 18-week BM engraftment of *Tet2*^Δ/+^ and *Tet2*^Δ/+^, *TNFr*^KO^ test cells in WT or *Jak2*^V617F^ host BM (n= 5 / group). (**E-H**) *Tet2*^Δ/+^ test cell (E) 16-week PB engraftment, (F) 18-week BM engraftment, (G) HSPC number and (H) HSC number chimeric mice receiving neutralizing antibodies against IL-12 and/or TNFα (n= 4–5 / group). **(I)** qPCR gene expression analysis of *Tet2*^Δ/+^ HSPCs from indicated treatments of genetic models depicting levels of indicated genes (normalized to non-treated controls; n = 2–3 / group). **(J-L)** Analysis of (J) spleen weight, (K) hematocrit and (L) modified read fold change for CRISPR engineered *TET2* mutations in cord blood CD34^+^ cells in treatment-control and IL-12-neutralizer treated PDX mice co-transplanted with PV UPN:702759 CD34^+^ cells (n= 2–3 / group). **(M)** Schematic depicting mechanism of PB cells providing a selective advantage to independent *TET2*-mutant clones through cytokine support. *p ≤0.05, **p ≤0.01, ***p ≤0.001, ****p ≤0.0001

With the identification of IL-12 and TNFα as potential selective pressures for independent *Tet2*-mutant clone expansion in parallel to MPN, we aimed to evaluate the functional consequences of disrupting these pathways. To enhance the clinical relevance, we replicated mutational burdens that more closely mirror those observed in MPN patients with input thresholds determined by the *Jak2*^V617F^ titration experiment. As such, chimeras utilized in functional studies were established by transplanting 1.5×10^6^ *Jak2*^V617F^ BM cells (CD45.2) with 4.5×10^5^ test BM (CD45.1/2) and 1.05×10^6^ WT support cells (CD45.1) into lethally irradiated recipients (CD45.1) to produce a starting fraction of test BM of approximately 15% in an environment composed of approximately 50% *Jak2*^V617F^-mutant host BM. First, to assess the implications of TNFα on the observed competitive advantage of *Tet2*-mutant clones, Vav-Cre;*Tet2*^fl/+^ mice were crossed with a TNFα-receptor genetic deletion model (p55/p75 germline knockout = “KO”) to establish mice wherein *Tet2*-mutant hematopoietic cells lack receptors for TNFα. To determine the effect of ameliorating TNFα signaling in *Tet2*-mutant cells, BM cells from either *Jak2*^V617F^ or WT mice were mixed with either *Tet2*^Δ/+^*, TNFαr*p55^−/−^p75^−/−^ (= “*TNFr*^KO^”), *Tet2*^Δ/+^/*TNFr*^KO^, or WT test BM and co-transplanted with WT support. Blood (Figure 5A–C) and BM analysis (Figure 5D) revealed the competitive advantage of *Tet2*-mutant cells in a *Jak2*^V617F^-mutant environment was significantly blunted by genetic deletion of TNFα receptors on the *Tet2*-mutant cells.

To evaluate this finding in a more translational system, we administered murine biosimilars of TNFα and IL-12 monoclonal neutralizing antibodies (adalimumab / ustekinumab respectively) into mouse chimeric models between weeks 4–10 post-transplant. Inhibition of TNFα and IL-12 dramatically reduced *Tet2*^Δ/+^ cell engraftment in a *Jak2*^V617F^-mutant background in the PB (Figure 5E), BM (Figure 5F) and HSPC populations (Figure 5G). Notably, neutralization of IL-12 and TNFα suppressed *Tet2*^Δ/+^ cells down to the HSC level – an effect most significantly achieved by IL-12 neutralization (Figure 5H). Gene expression analysis of *Tet2*^Δ/+^ HSPCs isolated from a *Jak2*^V617F^-mutant background at the end of the 18-week study from control and treated cohorts showed targeting the IL-12/TNFα axis normalized dysregulation in proliferation (*Mki67*) and self-renewal (*Hoxa10*, *Hlf, Fgd5*, and *Mecom*) pathways. Similar normalization of these abnormal gene expression programs was also observed in *Tet2*^Δ/+^ HSPCs genetically deficient for TNFα receptors (Figure 5I). Thus, genetic and pharmacological inhibition of aberrant MPN cytokine signaling abrogates the competitive advantage of *Tet2*^Δ/+^ HSPCs in the presence of *Jak2*^V617F^-mutant cells.

To evaluate the effect of IL-12 neutralization in a human system, we utilized a *JAK2*^V617F^-mutant PV patient sample that previously demonstrated support of parallel *TET2*-mutant clones (UPN:702759). 1×10^5^ CD34^+^ PV patient cells were co-transplanted with 2.0×10^4^ CB-derived CD34^+^ cells harboring gRNA-mediated *TET2* mutations. Between weeks 10–16 post-transplant, a human IL-12 neutralizing agent was administered to the treatment group. Control mice exhibited signs of disease progression characterized by increased spleen weight and decreased hematocrit, while mice receiving the IL-12 neutralizing agent retained PV-like pathologies (Figure 5J–K). Strikingly, IL-12 neutralization was able to mitigate the competitive advantage of *TET2-*mutant clones in the presence of PV patient cells (Figure 5L). These findings reveal that targeting IL-12 could be a potential therapeutic approach for restricting *TET2*-mutant CH in the background of an existing *JAK2*^V617F^-mutant driven MPN to minimize future risk of disease progression to pAML (Figure 5M).

These data establish that *JAK2*^V617F^-mutant cells potentiate parallel expansion of independent clones as a non-classical trajectory of disease progression in MPN. We show that MPN cells drive this parallel evolution through an IL-12/TNFα cytokine axis, which biases *Tet2*-mutant progenitors towards increased proliferation and myeloid differentiation. Genetic and pharmacological inhibition of IL-12 and TNFα resulted in both functional and molecular rescue of these phenotypes. Our findings provide crucial insights into how sAML which lacks the *JAK2*^V617F^ driver mutation can evolve from an antecedent *JAK2*^V617F^-mutant MPN. These results highlight a specific example of clonal dynamics and cell competition within a *JAK2*^V617F^-mutant context; however, it is important to acknowledge that clones with other CH mutations (e.g. *DNMT3A*) as well as MPNs driven by other mutations (*CALR*, *MPL*) may confer different patterns of clonal evolution. We aim to leverage these findings to enhance disease surveillance in MPN populations. With improved genomic monitoring, targeted interventional therapies to prevent the expansion of emerging pre-leukemic clones could reduce risk of parallel disease evolution in MPN, a significant therapeutic advantage for a substantial subset of patients.

## Materials and Methods

### Human Samples

All cord blood specimens were anonymized and no member of the study team had access to information that allowed the specimens to be linked to identifiable individuals. These studies were thus deemed “nonhuman studies” by the Washington University Human Research Protection Office. De-identified cord blood specimens were collected as part of a study approved by the Human Research Protection Office and the Institutional Review Board at Washington University School of Medicine (IRB# 202104011) after patients provided informed consent in accordance with the Declaration of Helsinki. De-identified bone marrow sample specimens were collected as healthy donor controls (IRB# 201103258). MPN patient samples were obtained according to a protocol approved by the Washington University Human Studies Committee (WU no. 01–1014). All patients previously provided consent to have samples banked and were not newly recruited for this study. Mutation information for patient samples was originally obtained from clinical testing with a targeted, error-corrected sequencing assay that covers 56 genes recurrently mutated in myeloid cancers. The assay has a documented limit of detection of 2% variant allele fraction (VAF) for new variants identified at initial diagnosis and 0.1% VAF for previously identified variants for molecular disease monitoring^42^. Mononuclear cells using SepMate-50 (StemCell Technologies #85450) mediated ficoll gradient extraction according to standard procedures. CD34+ cells were isolated using magnetic enrichment (Miltenyi Biotec #130-100-453) and cultured overnight in SFEMII media (StemCell Technologies #09605) supplemented with 50 U/mL penicillin-streptomycin (Fisher Scientific #MT30002CI), 50 ng/mL human stem cell factor (SCF; Miltenyi Biotec #130–096–695), 50 ng/mL human thrombopoietin (TPO; Miltenyi Biotec #130–094–013), and 50 ng/mL human FLT3L (Miltenyi Biotec #130–096–479). Enrichment efficiency was confirmed by flow cytometry.

### Single Cell DNA Sequencing and Analysis

Single-cell sequencing was performed using the Mission Bio Tapestri platform using methods similar to previous single-cell studies^43^.Cryovials of patient bone marrow or peripheral blood were thawed, resuspended in Hanks Balanced Salt Solution (HBBS, Corning #21021CV) and quantified by cellometer (Nexelom Bioscience). Cells were stained with 7AAD and live cells were sorted using a MoFlo cell sorter (Dako). One million live cells per sample were utilized for single-cell analysis on the Tapestri platform. Live cells were encapsulated into microfluidic droplets using the Tapestri instrument, lysed, and barcoded for library amplification using the Mission Bio myeloid panel (Table S2). This panel covers 45 genes that are recurrently mutated in myeloid malignancies. After library amplification the droplets were broken, and the libraries were isolated by Ampure XP bead cleanup (Beckman Coulter). Each purified library underwent PCR amplification using sequencing primers containing library-specific indexes followed by Ampure XP bead cleanup. Library quality was assessed using the TapeStation (Agilent Technologies) and Qubit (Thermo Fisher Scientific). Libraries were sequenced on the Illumina NovaSeq 6000 platform using the 300-cycle kit targeting 173 million read pairs for the DNA myeloid panel to ensure adequate coverage across the panel for each cell captured. FASTQ files containing the sequenced read information were analyzed using the cloud-based Tapestri Pipeline without modification. The pipeline trimmed the adapter sequences, mapped reads to the human reference genome (hg19) using BWA, and assigned each read to a unique cell. GATK v4/Haplotypecaller was used to call genotypes for each cell. Somatic mutations were retained if they were observed in >1% of cells and were not likely to arise due to allelic dropout. Sequencing results were further normalized, clustered, and annotated using the Mission Bio Mosaic version 2.4. Figures were generated using the Mission Bio Mosaic v2.4 pipeline.

### Droplet Digital PCR (ddPCR)

*JAK2*^V617F^ variant validation by ddPCR was performed on the QX200 platform (Bio-Rad) following similar methods as previously described^44^. Briefly, 20–50 ng of DNA was utilized per individual reaction. Droplets were generated using the QX200 droplet generator (Bio-Rad) following standard manufacturer protocols. PCR amplification of droplets was performed in a deep-well thermocycler (Bio-Rad) followed by analysis on the QX200 droplet reader (Bio-Rad).

### CRISPR and VAF Determination

Single guide RNAs (gRNA) targeting *TET2*, *TP53*, and *AAVS1* were designed using the UCSC Genome Browser. Multiple gRNAs were tested, the sequences for gRNAs (Synthego) used for final experimentation are as follows:

g*TET2*: GAAGCTACTGTGTTTGGTGC
g*TP53*: TGATGGTGAGGATGGGCCTC
g*AAVS1*: GGGGCCACTAGGGACAGGAT

Single stranded oligo donor nucleotides (ssODN) for *TET2*^R1216*^, *TP53*^R248Q^, and *AAVS1*^Silent^ were designed using the UCSC Genome Browser and were purchased from IDT and are as follows:

*TET2*R1216*:
  GGTTGGGGTGGGGGGTGTTTGGGATGGAATGGTGATCCACGCAGGTGGTTCGCAGAAGCAGCAGTGAAGAGAAGCTACTGTGTTTGGTGCGAGAGTGAGCTGGCCACACCTGTGAGGCTGCAGTGATTGTGATTCTCATCCTGGTGTGGGAAGGAATCCC
*TP53*^R248Q^:
  GCCAGTGTGCAGGGTGGCAAGTGGCTCCTGACCTGGAGTCTTCCAGTGTGATGATGGTGAGGATGGGCCTCTGGTTCATGCCGCCCATGCAGGAACTGTTACACATGTAGTTGTAGTGGATGGTGGTACAGTCAGAGCCAACCTAGGAGATAACACAGGC
*AAVS1*^Silent^:
  AATGTGGCTCTGGTTCTGGGTACTTTTATCTGTCCCCTCCACCCCACAGTGGGGCCACTAGGGACAGGTTTGGTGACAGAAAAGCCCCATCCTTAGGCCTCCTCCTTCCTAGTCTCCTGATATTGGGTCTAACCCCCACCTCC

Nucleofection was performed using a Neon Transfection System (Invitrogen #MPK5000) with the following parameters: Voltage: 1600, Width: 10, Pulses: 3. 48 hours post-nucleofection, approximately 100,000 cells were set aside to quantify CRISPR/Cas9 targeting efficiency using PCR amplicon-based deep sequencing. Libraries were sequenced with the Illumina MiSeq platform and output was analyzed using the CRISPREsso2 web-based software^45^. The following primer pairs were used to generate amplicons:

TET2 FWD: TGCAAGTGACCCTTGTTTTG
TET2 REV: ATTTCCTCAGCGTCTCGGTA
TP53 FWD: TGGAAGAAATCGGTAAGAGGTG
TP53 REV: TGGCTCTGACTGTACCACCA
AAVS1FWD: ACAGGAGGTGGGGGTTAGAC
AAVS1REV: CCCCTATGTCCACTTCA

### *Ex Vivo* Competition Assays

CD34+ cells were immunomagnetically isolated (Miltenyi Biotec #130-046-702) using the AutoMACS Neo system from PV or MF patient specimens and healthy donor BM and used as “host” cells. In parallel, CD34+ cells were isolated from umbilical cord blood obtained from healthy donors and used as “test” cells. Test cells were nucleofected with gRNA and ssODN templates targeting TET2, TP53, or AAVS1 as above. Host and test cells were combined at a ratio of 100,000 host cells to 20,000 test cells per well in a round-bottom 96-well plate and maintained in SFEMII media (StemCell Technologies #09605) supplemented with 50 U/mL penicillin-streptomycin (Fisher Scientific #MT30002CI), 50 ng/mL human stem cell factor (SCF; Miltenyi Biotec #130–096–695), 50 ng/mL human thrombopoietin (TPO; Miltenyi Biotec #130–094–013), and 50 ng/mL human FLT3L (Miltenyi Biotec #130–096–479) under normoxic conditions. Co-cultures were harvested on days 6 and 12 post-plating. At each time point, cell pellets were collected for genomic DNA extraction using PureLink gDNA column-based purification kit (Invitrogen #K182002). Targeted loci were amplified by PCR and amplicons were sequenced and analyzed as above enabling assessment of relative clonal expansion of gene-edited test cells within the mixed culture.

### Mice and Transplantation

The Institutional Animal Care and Use Committee at Washington University School of Medicine approved all animal procedures. Mice were housed in specific pathogen-free conditions at Washington University School of Medicine on a 12:12-hour light:dark cycle in temperature- and humidity-controlled rooms. Donor mice were typically 10–12-weeks old for experimentation. Both male and female mice were used. Mice used in murine competition experiments were all C57Bl/6 background. *Jak2*^V617F/+ 46^ and *Tet2*^fl/+ 17^ were crossed to Vav-Cre mice (The Jackson Laboratory #004682). *Tp53*^R172H/+ 47^ and *Tnfrsf1b*^*tm1Imx*^ *Tnfrsf1a*^*tm1Imx*^ (*Tnfr*^KO^; The Jackson Laboratory #003243) mice were germline knock-in and knock-out mutants respectively. Vav-Cre;*Tet2*^fl/+^ mice were crossed to *Tnfr*^KO^ to generate *Tet2*^Δ/+^*Tnfr*^KO^ mice. Recipient mice (C57Bl/6 CD45.1, The Jackson Laboratory #002014) were approximately 8-weeks old and transplanted by retro-orbital injection after a split dose (4-hours apart) of 10.5 Gy irradiation. For two-way chimeras, 2.5×10^6^ whole bone marrow (WBM) cells from *Jak2*^V617F^ or WT control (host, CD45.2) were transplanted along with 5×10^5^ WBM cells from either *Tet2*^Δ/+−^, *Tp53*^R172H/+^, or WT control (Test, CD45.1/2). For the *Jak2*^V617F^ titration experiment, a range of WBM (2.5×10^6^, 1.25×10^6^, 6.25×10^5^, 3.12×10^5^, 1.56×10^5^) sourced from *Jak2*^V617F^ (host, CD45.2) was transplanted with a range of WBM (1.25×10^6^, 1.88×10^6^, 2.19×10^6^, 2.34×10^6^, 2.50×10^6^) sourced from WT (support, CD45.1), and 5.0×10^5^ WBM cells sourced from *Tet2*^Δ/+^ (test, CD45.1/2). For three-way chimeras after the titration experiment, 1.5×10^6^ WBM cells from *Jak2*^V617F^ (host, CD45.2) was transplanted with 1.05×10^6^ WBM cells from WT (support, CD45.1) and 4.5×10^5^ WBM cells from *Tet2*^Δ/+^ (test, CD45.1/2). For TNF-receptor knock-out studies, chimeras were established with the same WBM cell numbers as in the three-way chimera experiments with *TNFr*^KO^ and *Tet2*^Δ/+^*Tnfr*^KO^ as test (CD45.2), *Jak2*^V617F^ or WT as host (CD45.1/2), and WT as support (CD45.1).

### PDX Transplantation

For patient derived xenograft (PDX) experiments, NOD-scid- *Il2rg*-null-3/GM/SF (NSGS; The Jackson Laboratory #013062) were used as recipients. 2.0×10^4^ cord-blood derived CD34^+^ cells from *AAVS1*, *TET2* and *TP53* nucleofected populations were transplanted with 1.0×10^5^ CD34^+^ cells derived from healthy donor (HD) human BM or PV or MF patient samples into sublethally irradiated (250 rads) 6–8-week-old NSGS mice via intra-tibial injections in a volume of 30 μL with 29-gauge U-100 insulin syringes (Covetrus #074076). For intra-tibial injections, mice were anesthetized with an intra-muscular injection of Ketamine/Xylazine mixture (2 mg/mouse; KetaVed, Vedco). The needle was inserted approximately 0.8 cm deep into the tibia and cells were gradually released into BM while the needle was gently removed.

### Cell Purification and Flow Cytometry of Cell Surface Markers

BM cells were isolated from iliac crests, femurs, and tibias of mice. Cells were stained in Hanks Balanced Salt Solution (HBBS, Corning #21021CV) containing 100 U/mL penicillin/streptomycin (Fisher Scientific #MT30002CI), 10 umol/L HEPES (Life Technologies #15630080) and 2% Serum Plus II (Sigma #14009C) at a density of 1.0 × 10^8^/mL. Staining was performed for >30 minutes at 4°C with desired antibodies. For cell sorting, BM was enriched with anti-mouse CD117-conjugated microbeads (Miltenyi Biotec #130–0910224) using the AutoMacs Neo Separator (Miltenyi Biotec), then stained with appropriate antibody cocktails. For HSC analysis and HSPC sorting from BM, the following antibodies were used: CD45.1-BV785 (clone A20; BioLegend #110743), CD45.2-BV605 (clone 104; BioLegend #109841), B220-APCcy7 (clone RA3–6B2; BioLegend #103224), Gr-1-APCcy7 (clone RB6–8C5; BioLegend #108424), Mac-1-APCcy7 (clone M1/70; BioLegend #101226), CD3e-APCcy7 (clone 145–2C11; BioLegend #100330), Ter119-APCcy7 (clone TER-119; BioLegend #116223), CD48-PECy7 (clone HM48–1; BioLegend #103424), CD150-PE (clone TC15–12F12.2; BioLegend #115904), c-Kit-BV421 (clone 2B8; BioLegend #105828), Sca1-APC (clone E13–161.7; BioLegend #122512).

For mouse peripheral blood analysis, blood samples were obtained by venipuncture. Red blood cells were lysed then samples were stained with the following antibodies: CD45.1-PE (clone A20; BioLegend #110708), CD45.2-BV421 (clone 104; BioLegend #109831), B220-PECy7 (clone RA3–6B2; BioLegend #103222), B220-APCCy7 (clone RA3–6B2; BioLegend #103224), Gr-1-PECy7 (clone RB6–8C5; BioLegend #108416), CD11b-PECy7 (clone M1/70; BioLegend #101216), CD3e-APCCy7 (clone 145–2C11; BioLegend #100330)

For PDX systems, the following antibodies were used for PB and BM sorting and analysis: anti-mouse CD45-BV605 (clone 30-F11; BioLegend #103139), anti-human CD45-APC (clone 2D1; BioLegend #368512), anti-human CD3-PEcy7 (clone HIT3a; BioLegend #300316), anti-human CD19-FITC (clone 4G7; BioLegend #392508), anti-human CD33-BV421 (clone WM53; BD #565949), anti-human CD34-PE (clone 561; BioLegend #343606), anti-human CD90-PECy7 (clone 5E10; BioLegend #328124), anti-human CD45RA-BV421 (clone HI100; BioLegend #304130), anti-human CD38-FITC (clone HB7; Invitrogen #11-0388-42), anti-human Lineage cocktail-BV510 (BioLegend #348807), and anti-human CD45-biotin (clone HI30; BioLegend #304004).

Dead cells were excluded from all analyses and downstream applications with 7AAD (BioLegend #420404, 1:100 dilution). Cell sorting was performed using MoFlo (Beckman Coulter). Flow cytometric analysis was performed using Northern Lights (CyTek). Acquired flow cytometry data were analyzed with FlowJo software.

### RNA Sequencing

7,000 donor-derived HSPCs (Lineage^−^ Sca-1^+^ c-Kit^+^ = “LSK”) from primary transplants were isolated using MoFlo (Beckman Coulter) from two pooled biological replicates from two independent cohorts (four total replicates per genotype per condition). Total RNA was extracted using NucleoSpin RNA Plus XS kit (Takara Bio #740990.50) and RNA Integrity was determined using Agilent Bioanalyzer. RNA with a RIN >8.0 was used to prepare for cDNA library with the SMARTer Ultra Low RNA kit for Illumina Sequencing (Takara-Clontech) per manufacturer’s protocol. Fragments were sequenced on an Illumina NovaSeq X Plus using paired end reads extending 150 bases.

### RNA Sequencing Analysis

Basecalls and demultiplexing were performed with Illumina’s DRAGEN and BCLconvert version 4.2.4 software with a maximum of one mismatch in the indexing read. RNA-seq reads were then aligned to the Ensembl release 101 primary assembly with STAR version 2.7.9a. Gene counts were derived from the number of uniquely aligned unambiguous reads by Subread:featureCount version 2.0.3. Sequencing performance was assessed for the total number of aligned reads, total number of uniquely aligned reads and features detected. Gene counts were imported into the R studio and used EdgeR and TMM packages to analyze normalization size factors to adjust for sample differences in library size. The matrix counts and TMM size factors were imported using Limma package. The mean-variance relationship of every gene and sample was then calculated and the count matrix was converted to log2 counts-per-million using Limma’s voom. Differential gene expressions (DEG) were analyzed using Limma with Benjamini-Hochberg false discovery rate. DEGs were defined by filtering for unadjusted *p*-value=0.05 and logFC=0.58 (equivalent to 1.5-fold change). Over-representation analysis of cancer-related pathways was performed using the NCI-Nature cancer pathways gene set library. Data visualization was performed using Enrichr and Appyters web-based software application package of meta Jupyter Notebook executions (volcano plot of ORA and UMAP of ORA) and MaGIC Volcano Plot Tool Software for volcano plot of DEGs^48,49^.

### pSTAT4 Intracellular Flow Cytometry

BM cells were stained for cell surface markers using the above makers in Cell Staining Buffer (BioLegend #420201) supplemented with dissolved Pierce Phosphatase Inhibitor tablets (Thermo Scientific #A32957). Following surface marker staining, whole BM was lysed and fixed with RBC Lysis/Fixation Solution (BioLegend #422401) at 37C for 15 minutes and subsequent permeabilization with True Phos Perm Buffer (BioLegend #425401) at −20°C for >60 minutes. After washing, the cells were resuspended at a concentration of 10×10^6^ cells per mL of buffer. 100uL of this cell solution was stained with anti-mouse pSTAT4-PE (clone A19016A; BioLegend #941206) at room temperature for 30 minutes in the dark. Flow cytometric analysis was performed using Northern Lights (CyTek). Acquired flow cytometry data were analyzed with FlowJo Software.

### Serum Cytokine Analysis

Peripheral blood samples were obtained by venipuncture and collected into serum separator tubes (BD Microtainer SST #365967). Samples were allowed to clot at room temperature for 30 minutes followed by centrifugation at 1,500g for 10 minutes at 4°C. Serum was stored at −80°C until analysis. Frozen serum aliquots were shipped to Eve Technologies Corporation (Calgary, AB, Canada) for multiplex cytokine analysis (Mouse Cytokine/Chemokine 32-Plex Discovery Assay Array or Human Cytokine/Chemokine 48-Plex Discovery Assay Array). Assays were performed according to the manufacturer’s protocols using a bead-based immunoassay (Luminex xMAP technology). Data were acquired via a Luminex 200 system, and analyte concentrations were calculated from standard curves generated with referenced cytokine standards.

### *in Vitro* Cytokine Assays

CD34^+^ cells were isolated from umbilical cord blood obtained from healthy donors and used as “test” cells. Test cells were nucleofected with gRNA and ssODN templates targeting *TET2* as above. 100,000 nucleofected cells were plated per well in a round-bottom 96-well plate and maintained in SFEMII media (StemCell Technologies #09605) supplemented with 50 U/mL penicillin-streptomycin (Fisher Scientific #MT30002CI), 100 ng/mL human stem cell factor (SCF; Miltenyi Biotec #130–096–695), 100 ng/mL human thrombopoietin (TPO; Miltenyi Biotec #130–094–013), and 100 ng/mL human FLT3L (Miltenyi Biotec #130–096–479) under normoxic conditions. Recombinant human IL-27 (PeproTech #200–38), IL1b (PeproTech #200–011B), IL-12 (PeproTech #200–12H), IL-1RA (PeproTech #200–01RA), or TNFα (PeproTech #300–01A) was added at concentrations of 0, 1, 25, 50, and 100 ng/mL each into separate wells containing CD34+ cells every 48 hours. Co-cultures were harvested on day 6 post-plating and cell pellets were collected for genomic DNA extraction using PureLink gDNA column-based purification kit (Invitrogen #K182002). Targeted loci were amplified by PCR and amplicons were sequenced and analyzed as above enabling assessment of relative clonal expansion of gene-edited test cells tested at each cytokine and each concentration.

### *in vivo* Cytokine Neutralization

Mouse neutralizing InVivoMAb antibodies against interleukin-12 (Bio X Cell #BE0052) and TNFα (Bio X Cell #BE0058) and the human neutralizing InVivoSIM antibody against interleukin-12 (Bio X Cell #SIM0020) were administered *in vivo*. Mice were randomized into treatment groups based on test cell peripheral blood engraftment. Neutralizing antibodies were administered intraperitoneally at 500ug per injection twice per week (cumulative weekly dose of 1mg) for a total treatment duration of 5-weeks during each transplant period as adopted and modified from prior studies^50^. Control animals received isotype-matched antibodies following the same dosing schedule. All injections were performed under aseptic technique with mice closely monitored for signs of distress throughout the study period.

### Quantitative Real-time PCR

HSPCs (Lineage^−^ Sca-1^+^ c-Kit^+^ = “LSK”) were isolated using MoFlo (Beckman Coulter) from three pooled biological replicates. Total RNA was extracted using NucleoSpin RNA Plus XS kit (Takara Bio #740990.50). cDNA was synthesized using SuperScript VILO cDNA kit (ThermoFisher Scientific #1174050) and diluted 1:10 for real-time qPCR assays. Diluted cDNA samples were used for qPCR with Taq Man Master Mix (ThermoFisher Scientific #4304437), probes, and 18S (internal control). qPCR was performed using a Step One Plus Real-Time qPCR machine. The following probes (ThermoFisher Scientific #4331182) were used for the assay: *Hoxa10* (Mm00433966_m1), *Hlf* (Mm00723157_m1), *Fgd5* (Mm00554954_m1), *Mecom* (Mm00491303_m1), *Mki67* (Mm01278617_m1), *S100a8* (Mm00496696_g1), and *S100a9* (Mm00656925_m1).

### Statistical Analysis

One-way ANOVA with Tukey correction for multiple comparisons (multiple groups) or unpaired two-tailed t-test (two groups) were used for statistical comparison where appropriate. Significance is indicated using the following convention: *p ≤0.05, **p ≤0.01, ***p ≤0.001, ****p ≤0.0001. All graphs represent mean ± s.e.m. unless otherwise indicated.

## Supplementary Material

Supplementary Files

This is a list of supplementary files associated with this preprint. Click to download.

- SUPPNaturepAML11.11.25.pdf
- DataS1.xlsx

Figures S1 to S8

Table S1 to S2

Data S1

## Data Availability

Data not available in the main text or the supplementary materials are available through request to the corresponding author. All raw read data (FASTQ files) for RNA sequencing are publicly available at the Gene Expression Omnibus database accession number GSE308233.
